# Metabolic health and cardiovascular risk among Cameroonian adults with and without obesity

**DOI:** 10.1371/journal.pgph.0006870

**Published:** 2026-09-23

**Authors:** Maxwell Wandji Nguedjo, Ousmane Mfopou Mbiondi, Kevin Fabrice Paul Mandeng, Alex Dimitri Tchuenchieu Kamgain, Gabriel Nama Medoua, Judith Laure Ngondi, Julius Enyong Oben

**Affiliations:** 1 Centre for Food, Food Security and Nutrition Research, Institute of Medical Research and Medicinal Plant Studies, Yaounde, Cameroon; 2 Department of Public Health, Faculty of Medicine and Biomedical Sciences, University of Yaounde 1, Yaoundé, Cameroon; 3 Department of Biochemistry, Faculty of Sciences, University of Yaounde 1, Yaounde, Cameroon; 4 Department of Biotechnology and Food Technology, University of Johannesburg, Johannesburg, South Africa; Universiti Malaya, MALAYSIA

## Abstract

Obesity contributes to increased morbidity and mortality, yet its metabolic heterogeneity remains underexplored in sub-Saharan Africa. This study examined associations between body composition, insulin sensitivity, inflammation, and the metabolically healthy profile (MHP), and assessed cardiovascular risk among Cameroonian adults with and without obesity. In this cross-sectional study, 490 participants from the West region of Cameroon underwent anthropometric and metabolic assessments. Ten-year cardiovascular risk was estimated using the Framingham risk score. Logistic regression identified predictors of MHP and determinants of elevated cardiovascular risk. The prevalence of MHP was 59.6% among individuals with normal weight, 11.5% among those with overweight, and 38.2% among those with obesity. Participants with overweight, obesity, android obesity or insulin resistance were 92%, 59%, 58%, and 46% less likely to have a MHP than participants in the control groups, respectively. Inflammation defined solely by CRP-hs was not predictive. Compared with metabolically unhealthy counterparts, metabolically healthy obese (OR = 6.27; 95% CI: 3.15-12.48) and overweight (OR = 2.50; 95% CI: 0.72-8.65) individuals had substantially lower predicted 10-year cardiovascular risk. Metabolic phenotyping reveals that not all Cameroonians with overweight and obesity present the same cardiovascular risk. Incorporating fat distribution and insulin sensitivity into clinical evaluation could improve early identification of high-risk individuals and guide tailored prevention strategies.

## Introduction

Overweight or obesity has become a real public health problem, since the increase in its prevalence in both rich and poor countries is undeniable and worrying [1]. According to the World Health Organisation (WHO), there are currently an estimated 1.9 billion overweight adults and almost 650 million obese people worldwide [2]. Obesity is frequently associated with many cardio-metabolic abnormalities such as arterial hypertension, insulin resistance, and dyslipidaemia, which provide the basis for type 2 diabetes and cardiovascular disease [3]. However, the frequency of these abnormalities varies considerably among obese people, making this chronic disease a very heterogeneous clinical situation [4]. Some people have a metabolically healthy profile (MHP) despite a high body mass index (BMI) [5]. People with this MHP may be less at risk of developing the health problems mentioned above than people with a metabolically unhealthy profile [6]. One study found that high BMI was responsible for 4.0 million deaths worldwide, and around 40% of these deaths occurred in people who do not have obesity [7]. Thus, the individual risk of developing obesity-related comorbidities varies considerably, which could be explained by the degree of adiposity [7]. Body composition could therefore contribute to understanding the increased cardiovascular risk observed in people with a metabolically unhealthy profile [8]. Research has also shown that subjects with overweight or obesity display differences in the amount of excess fat they accumulate, in the anatomical distribution of fat, and their metabolic profile [9]. Other criteria, such as insulin resistance and high-sensitivity C-reactive protein (hs-CRP), have also been proposed better to characterise the metabolically healthy profile of individuals with obesity [8].

In Cameroon, the overall prevalence of obesity was 15.1% between 2000 and 2017 according to the body mass index (BMI) [10]. Moreover, it is estimated that this prevalence will double over a decade [11]. Cameroon is a particularly relevant context for the study of metabolic phenotypes of obesity due to the rapid nutritional transition observed in recent years, the continuous progression of obesity and cardiometabolic diseases, as well as the still limited number of local data on the physiopathological determinants of metabolic health. Moreover, the country is characterized by a high level of ethnic diversity, with many population groups spread across different regions and showing differences in dietary habits, lifestyles, and health-related behaviours [12]. Despite this diversity, local data on the physiopathological determinants of metabolic health remain limited. These findings underscore that the obesity epidemic is a growing component of the non-communicable disease burden and may worsen in the absence of appropriate preventive and therapeutic measures [13]. Scientific data on the metabolic profile of people with overweight and obesity are therefore needed to develop an effective management strategy. Research by Yangoua et al. [14] found that nearly half of Cameroonians with obesity did not have insulin resistance. To our knowledge, no study has specifically explored the relationships between body composition, insulin sensitivity, inflammation, and metabolically healthy phenotype in the Cameroonian population. This raises the question of whether stratification of people with overweight or obesity according to their metabolic status could offer new perspectives for personalized management. To answer this question, the present study aims to perform a detailed metabolic phenotyping of individuals with overweight or obesity in order to better understand the pathophysiological relationships between overall adiposity, body fat distribution, insulin status, the inflammatory status, and metabolically healthy phenotype, as well as to identify individuals at high cardiovascular risk among Cameroonian adults with and without obesity.

## Materials and methods

### Ethics statement

This study has received prior approval from the National Ethics Committee for Human Health Research of Cameroon under the number 2014/08/488/CE/CNERSH. The study was conducted in strict compliance with medical ethics in accordance with the principle described in the declaration of Helsinki. Approval from local authorities was granted following the submission of the ethical approval and the participants’ information leaflet. After being informed of the objectives of the study, the voluntary participants provided their written consent. The information notice specified the purpose of the study, the confidentiality of the study, the benefits of the study, minimized risks, and the right to voluntarily participate or withdraw from the study at any time.

### Study design and population

This cross-sectional study was conducted for three months, from 4 May 2015–30 June 2015, in three semi-urban cities of the western region of Cameroon. The data collection sites included the cities of Bafoussam, Dschang, and Bangangté. The study population consisted of Cameroonian adults regardless of gender. Participants were adults aged 21 years or older, residing in the study area, with no medical contraindications to participation. Individuals undergoing medication or therapy likely to alter metabolism, as well as those diagnosed with a cardiometabolic disease before the start of the study, were excluded to avoid any influence on metabolic parameters.

### Sampling procedure

Participants were recruited through the organisation of cardiovascular disease screening campaigns in the target population using a convenience sampling design. The sample size was determined using Magnani’s [15] formula, taking into account the estimated prevalence of metabolic syndrome (14%) in semi-urban areas in Cameroon [16], the 5% margin of error, and the 95% confidence level. Although the minimum required sample size was 185, we over-recruited (962 participants) to improve statistical power and account for exclusions. After applying eligibility criteria, 490 participants remained for analysis.

### Anthropometric measurements and blood pressure

Height was determined using a Harpended^TM^ stadiometer accurate to the centimetre. Weight was assessed in participants with average clothing and bare feet, using a Tanita^TM^ scale accurate to the kilogram. Body mass index (BMI) was calculated by dividing weight (kg) by height squared (m^2^) and expressed in kg/m^2^. Waist circumference and hip circumference were measured to the nearest centimetre using a soft, non-stretch measuring tape. The waist-to-hip circumference ratio was calculated for each participant.

Blood pressure was determined using an OMRON electronic radial sphygmomanometer applied to the participant’s left arm in the seated position, after at least 10 minutes’ rest. Two successive measurements were taken 15 minutes apart. The first measurement was taken after a 10-minute rest in a seated position, and the second was taken at the end of the interview. The average of these two measurements was used to assess the presence or absence of high blood pressure in the participant.

### Biological data and laboratory tests

Blood was drawn in the morning from fasting participants after an 8-hour fasting period. Approximately 5 mL of blood was taken by venipuncture from the elbow and collected in EDTA tubes. The samples were centrifuged at 4500 g for 10 minutes. The plasma obtained was stored at -20°C in 200 μL aliquots for biochemical testing.

Total cholesterol and plasma triglycerides were assessed by enzymatic spectrophotometric methods using the ChronoLab Kit. Fasting blood glucose levels were measured directly on the participant’s finger using a glucometer (One-touch plus) with reagent strips, using the glucose oxidase method. The concentrations of insulin and high-sensitivity C-reactive protein (CRP-hs) were quantified using the sandwich ELISA (Enzyme-linked immunosorbent assay) technique.

### Variable grouping

The criteria of the World Health Organisation [17] were used to classify participants according to their nutritional status: normal weight (BMI of 18.5-24.9 kg/m^2^), overweight (BMI of 25-29.9 kg/m^2^), and obesity (BMI ≥ 30 kg/m^2^).

Fat localisation was determined by the waist-to-hip circumference ratio as follows: a waist-to-hip circumference ratio ≥ 0.90 in men or ≥ 0.85 in women characterises android obesity, while a waist-to-hip ratio < 0.90 in men or < 0.85 in women characterises gynoid [18].

Participants’ insulin status was categorised into two groups: insulin sensitivity (HOMA-IR<2.6) and insulin resistance (HOMA-IR ≥ 2.6). Homeostasis Model Assessment for Insulin Resistance (HOMA-IR) was calculated using the formula described by Matthews et al. [19]: HOMA-IR = fasting insulin (IU/mL) × fasting plasma glucose (mM)/22.5.

The inflammatory status of the participants was categorized into two groups according to the level of high-sensitivity C-reactive protein (CRP-hs): absence of inflammation (CRP-hs < 3mg/L) and inflammation (CRP-hs ≥ 3mg/L), following the criteria of Widman et al. [20].

To assess the metabiolic status of the participants, some criteria were selected. These criteria included the following metabolic abnormalities: systolic blood pressure (SBP) ≥ 130 mmHg and/or diastolic blood pressure (DBP) ≥ 85 mmHg; fasting blood glucose ≥ 100 mg/dL; total cholesterol ≥ 200 mg/dL, and triglycerides ≥ 150 mg/dL. The thresholds of these criteria are based on the work of Nguedjo et al. [21] conducted in a Cameroonian context, as well as the International Diabetes Federation (IDF) modified according to Fezeu et al. [22]. According to these authors, there is no need to reclassify the thresholds for participants. Participants were considered to have a metabolically healthy profile in the absence of any metabolic abnormalities, while a metabolically unhealthy profile was defined by the presence of at least one of the four metabolic abnormalities mentioned above.

### Prediction of a cardiovascular event

The prediction of 10-year cardiovascular risk in metabolically healthy and metabolically unhealthy subjects was determined using the slightly modified Framingham Risk Score (FRS) model (high-density lipoprotein cholesterol has been replaced by triglycerides, and the treatment of hypertension by high diastolic blood pressure). The calculation of the Framingham score took into account factors such as sex, age, total cholesterol, triglycerides, systolic blood pressure, high diastolic blood pressure, the presence or absence of diabetes (blood glucose≥126 mg/dL), and smoking habits. From the obtained score values, 10-year cardiovascular risk was estimated as a percentage and then classified into three categories: low CRV (FRS < 10%), moderate CRV (FRS: 10–20%), and high CRV (FRS > 20%) [23]. However, in order to better identify individuals requiring more intensive preventive care, we have grouped these categories into two levels: low to moderate CRV (FRS ≤ 20%) and high CRV (FRS > 20%).

### Data analysis

The data were entered and checked using Excel version 2013. Statistical analyses were performed using SPSS (Statistical Package for the Social Sciences) version 25.0 for Windows. Results were expressed as mean ± standard error (SE) and percentage (%). The Student’s t-test was used to detect differences in means between two groups of a continuous dependent variable. P values <0.05 were considered statistically significant. Bivariate logistic regression analyses were used to estimate Odd ratios (OR) and their 95% confidence intervals (CI). The estimate of the relative decrease in the chances of the metabolically healthy profile appearing was obtained according to the probability reduction formula (Probability reduction (%) = (1−OR) ×100) when the odds ratio < 1.

## Results

### Clinical and paraclinical characteristics according to the metabolic profile and by category of body mass index in the study population

The analysis of Table 1 reveals that subjects in good metabolic health were more frequent among individuals with a normal weight (59.6%), followed by those with obesity (38.2%), and those with overweight (11.5%). In subjects of normal weight, age, blood pressure, triglycerides, total cholesterol, blood glucose, insulin, HOMA-IR, and highly sensitive C-reactive protein were significantly lower in metabolically healthy individuals than in metabolically unhealthy individuals (p < 0.05). In overweight individuals, values for diastolic pressure, BMI, triglycerides, blood glucose, insulin, and HOMA-IR were also lower in metabolically healthy subjects (p < 0.05). Finally, in obese individuals, age, blood pressure, triglycerides, cholesterol, blood glucose, HOMA-IR, and highly sensitive C-reactive protein showed the same significant differences, confirming more favorable cardiometabolic profiles in healthy individuals.

**Table 1:** Clinical and para-clinical characteristics according to the metabolic profile among participants with normal weight, overweight, and obesity.

Parameters	Normal			Overweight			Obesity		
	Metabolically Healthy	Metabolically Unhealthy	P-value	Metabolically Healthy	Metabolically Unhealthy	P-value	Metabolically Healthy	Metabolically Unhealthy	P-value
Size (percentage)	99(59.6)	67(40.4)	0.013	12(11.5)	92(88.5)	<0.001	84(38.2)	228(61.8)	<0.001
Age (years)	30.58 ± 0.75	36.75 ± 2.45	0.006	48.67 ± 0.56	48.39 ± 1.24	0.937	40.14 ± 1.18	48.44 ± 1.10	<0.001
SBP (mmHg)	116.71 ± 0.82	130.84 ± 0.66	<0.001	113.00 ± 0.42	121.30 ± 1.67	0.078	116.14 ± 1.30	127.85 ± 1.85	<0.001
DBP (mmHg)	69.45 ± 0.40	85.55 ± 1.00	<0.001	58.00 ± 2.13	78.39 ± 1.46	<0.001	57.62 ± 0.68	75.53 ± 1.29	<0.001
BMI (Kg/m^2^)	21.86 ± 0.11	22.21 ± 0.16	0.061	28.02 ± 0.29	28.73 ± 0.07	0.461	34.20 ± 0.36	33.70 ± 0.33	0.337
TG (mg/dL)	100.32 ± 2.34	150.15 ± 2.76	<0.001	98.94 ± 0.75	148.62 ± 7.54	0.020	99.47 ± 3.50	121.97 ± 5.35	0.003
TC (mg/dL)	139.28 ± 3.26	188.18 ± 2.03	<0.001	119.39 ± 6.70	158.13 ± 8.57	0.109	138.65 ± 3.30	220.58 ± 6.40	<0.001
Glucose (mg/dL)	80.90 ± 1.00	109.83 ± 1.42	<0.001	88.33 ± 0.71	114.04 ± 3.63	0.003	89.76 ± 0.54	123.73 ± 2.63	<0.001
Insulin (µUI/mL)	7.67 ± 0.16	13.73 ± 0.17	<0.001	11.20 ± 0.52	20.23 ± 3.63	<0.001	12.34 ± 0.77	14.31 ± 0.61	0.046
HOMA-IR	0.71 ± 0.01	1.75 ± 0.04	<0.001	3.15 ± 0.17	5.56 ± 0.82	<0.001	2.75 ± 0.17	4.42 ± 0.20	<0.001
CRP-hs (mg/L)	1.69 ± 0.05	3.59 ± 0.10	<0.001	3.90 ± 0.24	4.11 ± 0.14	0.621	4.35 ± 0.19	5.10 ± 0.18	0.009

SBP: systolic blood pressure, DBP: diastolic blood pressure, TG = Triglycerides, TC = Total cholestérol, BMI = Body mass index, CRP-hs: highly sensitive C-reactive protein; HOMA-IR: Homeostasis Model Assessment for Insulin Resistance.

### Clinical and paraclinical characteristics according to the metabolic profile and by location of body fat among participants with overweight and obesity

The results in Table 2 indicate that gynoid obesity was more common than android obesity among people with overweight/obesity (41.4% vs. 23.1%). In those who had gynoid obesity, waist-to-hip ratio, diastolic pressure, triglycerides, total cholesterol, blood glucose, and highly sensitive C-reactive protein were significantly lower in metabolically healthy subjects than metabolically unhealthy subjects (p < 0.05). Similarly, in individuals with android obesity, blood pressure, triglycerides, cholesterol, blood glucose, and HOMA-IR were also reduced in metabolically healthy subjects (p < 0.05).

**Table 2:** Clinical and paraclinical characteristics according to the metabolic profile and by location of body fat among participants with overweight/obesity.

Parameters	Gynoid obesity		P-value	Android obesity		P-value
	Metabolically Healthy	Metabolically Unhealthy		Metabolically Healthy	Metabolically Unhealthy	
Size (percentage)	48(41.4)	68(58.6)	0.063	48(23.1)	228(76.9)	<0.001
waist-to-hip ratio	0.76 ± 0.01	0.82 ± 0.02	<0.001	0.94 ± 0.02	0.96 ± 0.01	0.478
SBP (mmHg)	116.94 ± 2.40	117.24 ± 1.53	0.816	113.83 ± 1.73	125.29 ± 1.28	<0.001
DBP (mmHg)	55.58 ± 0.63	69.88 ± 2.21	<0.001	59.75 ± 1.06	79.58 ± 0.93	<0.001
Triglycerides (mg/dL)	81.56 ± 4.18	110.13 ± 7.63	0.004	117.25 ± 2.62	142.32 ± 5.34	0.012
Total cholestérol (mg/dL)	127.92 ± 5.06	175.61 ± 10.76	0.001	144.57 ± 3.08	203.79 ± 6.32	<0.001
Glucose (mg/dL)	89.67 ± 0.66	118.82 ± 3.65	<0.001	89.50 ± 0.71	120.25 ± 2.42	<0.001
Insulin (µUI/mL)	14.23 ± 1.34	12.41 ± 0.66	0.188	13.67 ± 1.26	13.38 ± 0.55	0.810
HOMA-IR	3.18 ± 0.30	3.45 ± 0.18	0.432	3.02 ± 0.27	4.10 ± 0.20	0.008
CRP-hs (mg/L)	3.38 ± 0.18	4.35 ± 0.12	<0.001	4.85 ± 0.17	5.21 ± 0.21	0.294

SBP: systolic blood pressure, DBP: diastolic blood pressure, CRP-hs: highly sensitive C-reactive protein; HOMA-IR: Homeostasis Model Assessment for Insulin Resistance

### Clinical and paraclinical characteristics according to the metabolic profile and by insulin status among participants with overweight/obesity

Table 3 shows that people with overweight/obesity with insulin sensitivity were more represented than those with insulin resistance (45.8% vs. 31.6%). In insulin-sensitive subjects, blood pressure, triglycerides, cholesterol, and blood glucose were significantly lower in metabolically healthy individuals than in metabolically unhealthy individuals (p < 0.05). Similarly, in insulin-resistant individuals, blood pressure, cholesterol, and blood glucose showed lower levels in metabolically healthy (p < 0.05).

**Table 3:** Clinical and paraclinical characteristics according to metabolic profile and insulin status among participants with overweight/obesity.

Parameters	Sensitive insulin		P -value	Resistant insulin		P- value
	Metabolically Healthy	Metabolically Unhealthy		Metabolically Healthy	Metabolically Unhealthy	
Size (percentage)	44(45.8)	52(54.2)	0.009	72(31.6)	156(68.4)	<0.001
SBP (mmHg)	119.25 ± 1.05	127.03 ± 2.01	<0.001	119.25 ± 1.05	127.03 ± 2.01	0.022
DBP (mmHg)	56.67 ± 0.66	80.28 ± 1.18	<0.001	58.67 ± 1.11	73.88 ± 1.42	0.001
Triglycerides (mg/dL)	85.10 ± 5.46	145.69 ± 9.98	<0.001	111.51 ± 2.15	126.73 ± 4.61	0.062
Total cholestérol (mg/dL)	127.08 ± 5.75	191.68 ± 11.47	<0.001	144.00 ± 2.49	197.09 ± 6.13	<0.001
Glucose (mg/dL)	86.55 ± 0.61	107.17 ± 3.02	<0.001	92.15 ± 0.51	125.67 ± 2.46	<0.001
Insulin (µUI/mL)	7.19 ± 0.25	6.60 ± 0.30	0.170	19.67 ± 1.20	16.09 ± 0.45	0.010
HOMA-IR	1.52 ± 0.04	1.60 ± 0.05	0.292	4.44 ± 0.25	4.97 ± 0.16	0.101
CRP-hs (mg/L)	3.95 ± 0.21	4.04 ± 0.20	0.804	4.58 ± 0.24	5.00 ± 0.15	0.170

SBP: systolic blood pressure, DBP: diastolic blood pressure, CRP-hs: highly sensitive C-reactive protein; HOMA-IR: Homeostasis Model Assessment for Insulin Resistance.

### Clinical and paraclinical characteristics according to the metabolic profile and by inflammatory status among participants with overweight/obesity

The results in Table 4 indicate that individuals with overweight/obesity with insulin sensitivity were slightly more represented than those with insulin resistance (20.8% vs. 19.3%). In subjects without inflammation, blood pressure, triglycerides, cholesterol, and blood glucose were significantly lower in metabolically healthy people than metabolically unhealthy people (p < 0.05). Similarly, in people with inflammation, blood glucose, triglycerides, cholesterol, blood sugar, and HOMA-IR, lower levels were also observed in metabolically healthy subjects (p < 0.05).

**Table 4:** Clinical and paraclinical characteristics according to metabolic profile and by inflammatory status among participants with overweight/obesity.

Parameters	Absence of inflammation		P -value	Inflammation		P-value
	Metabolically Healthy	Metabolically Unhealthy		Metabolically Healthy	Metabolically Unhealthy	
Size (percentage)	20(20.8)	76(79.2)	0.003	44(19.3)	184(80.7)	<0.001
SBP (mmHg)	111.00±1.58	129.36±3.49	0.001	117.11±1.40	122.11±1.08	0.002
DBP (mmHg)	62.00±1.17	82.27±1.90	<0.001	56.53±6.20	75.35±1.09	<0.001
Triglycerides (mg/dL)	89.35±10.46	146.53±12.93	0.007	102.05±2.69	129.42±4.60	<0.001
Total cholestérol (mg/dL)	118.58±9.10	197.27±10.79	<0.001	140.89±2.85	194.94±6.36	<0.001
Glucose (mg/dL)	92.40±1.10	119.27±6.33	0.006	88.64±0.51	119.96±2.00	<0.001
Insulin (µUI/mL)	12.37±1.27	10.64±1.57	0.490	14.37±1.10	13.68±0.38	0.454
HOMA-IR	2.87±0.31	3.18±0.51	0.693	3.16±0.24	4.08±0.14	0.001
CRP-hs (mg/L)	2.22±0.06	2.19±0.05	0.396	4.87±0.15	5.29±0.12	0.057

SBP: systolic blood pressure, DBP: diastolic blood pressure, CRP-hs: highly sensitive C-reactive protein; HOMA-IR: Homeostasis Model Assessment for Insulin Resistance.

### Predictive factors of the metabolically healthy profile in the study population

Table 5 presents the associations between characteristics of adiposity, insulin status, inflammatory status and metabolically healthy profile. Taking as reference categories normal-weight individuals, those with gynoid obesity, insulin sensitivity, and no inflammation, participants with overweight (OR = 0.08; 95% CI: 0.04-0.17) and those with obesity (OR = 0.41; 95% CI: 0.27-0.63) had a significantly lower probability of having a metabolically healthy profile, corresponding to a decrease of about 92% and 59%, respectively, in the chances of being metabolically healthy. Similarly, participants with android obesity (OR = 0.42; 95% CI: 0.26-0.69) or insulin resistance (OR = 0.54; 95% CI: 0.33-0.88), were 58% and 46% less likely to have a metabolically healthy profile than their baseline counterparts, respectively. By contrast, no statistically significant association was observed between the presence of inflammation and the metabolically healthy profile (OR = 0.90; 95% CI: 0.50-1.64).

**Table 5:** Association between overall adiposity, fat localization, insulin status, inflammatory status, and metabolically healthy profile in the study population.

Parameters	Odds ratio (95% CI)	p-value
**Body mass index**		
Normal	1	<0.001 <0.001
Overweight	0.08 (0.04-0.17)	
Obesity	0.41 (0.27-0.63)	
**Fat localization**		
Gynoïd obesity	1	0.001
Androïd obesity	0.42 (0.26-0.69)	
**Insulin status**		
Sensitive insulin	1	0.015
Resistant insulin	0.54 (0.33-0.88)	
**Inflammatory status**		
Absence of inflammation	1	0.751
Inflammation	0.90 (0.50-1.64)	

OR: Odds ratio, CI: Confidence interval.

### Prediction of cardiovascular risk over 10 years in the population based on metabolic profile and body mass index category

Table 6 shows that in the same BMI category, the shift from a metabolically healthy to an unhealthy profile results in a significant increase in the risk of developing cardiovascular disease over the next 10 years. Thus, individuals with obesity and metabolically healthy had a 10-fold lower risk that individuals with obesity metabolically unhealthy, while individuals with overweight metabolically healthy had an approximately 9-fold lower risk than their metabolically unhealthy counterparts.

**Table 6:** 10-year cardiovascular risk prediction in the population based on metabolic profile among participants with normal weight, overweight, and obesity.

Metabolic profile	Odds ratio (95% CI)	P-value
Metabolically healthy normal weight	1	
Metabolically healthy overweight	2.50 (0.72-8.65)	0.148
Metabolically healthy obesity	6.27 (3.15-12.48)	<0.001
Metabolically unhealthy normal weight	8.26 (4.24-16.07)	<0.001
Metabolically unhealthy overweight	11.13 (5.67-21.84)	<0.001
Metabolically unhealthy obesity	16.33 (8.55-31.20)	<0.001

OR: Odds ratio, CI: Confidence interval.

## Discussion

The results of this study highlight the complexity of overweight and obesity within the Cameroonian population, taking into account overall adiposity, body fat distribution, as well as insulin and inflammatory status. It was found that body mass index (BMI) was essential for predicting the metabolically healthy profile in the study population. Indeed, the proportion of individuals with a metabolically healthy profile was higher among those with normal BMI (59.6%). Conversely, the increase in BMI was associated with higher levels of CRP-hs, plasma insulin, and blood glucose. These results are similar to those observed in a Chinese cohort [24]. These results suggest that the increase in adiposity is associated with a less favorable metabolic profile. However, this association is influenced not only by the amount of adipose tissue, but also by its distribution and functional characteristics [9]. Moreover, our results showed that the reduction in the chance of presenting a metabolically healthy phenotype was higher among individuals with overweight (92%) than those with obesity (59%), indicating that BMI alone is not sufficient to predict the metabolic profile since individuals with normal BMI may develop metabolic alterations. This observation is consistent with the results of López-Herrera et al. [25], who noted the existence of the metabolically unhealthy phenotype in adults with normal weight, overweight, and obesity in a Colombian population.

Beyond overall adiposity, the distribution of adipose tissue exerts a decisive influence on the metabolic profile. In this study, the waist/hip ratio allowed to distinguish gynoid accumulation from android accumulation. The results show that the gynoid distribution, associated with a lower waist/hip ratio, predicts a metabolically healthy profile more than the android distribution. This result is in line with the work of Peppa et al. [26], who showed that metabolically unhealthy obese individuals had higher abdominal accumulation and a higher waist/hip ratio than their healthy counterparts. Excess abdominal fat thus appears as a marker of dysfunctional adipose tissue, linked to increased lipolysis and gene expression changes involved in lipid storage and inflammation [27]. Conversely, gynoid accumulation appears to confer a cardioprotective effect due to higher adiponectin levels, better insulin sensitivity, and favorable lipid profile, linked to increased lipoprotein lipase activity and low lipolytic activity [28]. However, some people overweight/obesity exhibited an android distribution despite a metabolically healthy profile, this paradox being explained by the compartmentalization of abdominal fat, where deep subcutaneous adipose tissue, thicker than superficial, promotes metabolic alterations independently of other indicators of adiposity [9].

This study also showed that insulin sensitivity is a key marker of a metabolically healthy profile. However, some people with overweight/obesity had insulin resistance despite a favorable metabolic state. Similar results were reported by Hoddy et al. [29], indicating that individuals with obesity, whether metabolically healthy or unhealthy, exhibited reduced insulin sensitivity, although triglyceride levels, glucose, and insulin are lower in metabolically healthy subjects. The underlying mechanisms are still poorly understood, but McLaughlin et al. [30] showed that obese insulin-sensitive individuals expressed the PPARγ gene more in subcutaneous adipose tissue. In addition, the administration of PPARγ agonists (pioglitazone) to obese insulin-resistant individuals promoted the formation of small insulin-sensitive adipocytes, thus improving the metabolic profile. These observations suggest that an increase in PPARγ expression and adiponectin secretion could explain why some individual’s overweight/obesity maintain a metabolically healthy profile despite a high HOMA-IR index.

Inflammatory status also appears as a key element of the metabolic profile. In our study, nearly two-thirds of metabolically healthy overweight or obese individuals exhibited inflammation defined by elevated CRP-hs levels. The presence of low-intensity chronic inflammation in some metabolically healthy individuals may reflect an intermediate phase before metabolic abnormalities appear. In this context, the underlying physiological alterations could already be initiated, but remain insufficiently developed to be highlighted in a cross-sectional study.These results are consistent with those of Iglesias et al. [31], who observed comparable levels of CRP-hs in metabolically healthy and unhealthy obese. Association analysis showed that inflammation was not a predictor of the metabolically healthy profile. The lack of association between CRP-hs and the metabolically healthy phenotype observed in our study could be explained by the non-specific nature of this biomarker. Indeed, CRP-hs reflects an overall systemic inflammation and its concentrations can be influenced by various factors independent of metabolic alterations, including diet, physical activity, and genetics [32]. This observation is in line with the conclusions of Langenberg et al. [33], according to which inflammatory markers only contribute weakly to explaining the link between metabolic syndrome and cardiovascular mortality, their predictive value remaining uncertain.

Our study showed that a lower BMI, regardless of the obesity phenotype, was accompanied by a decrease in cardiovascular risk over 10 years. Similar results were observed by Wang et al. [34] in a Chinese cohort, where metabolically healthy obese had an increased risk of CVD compared to metabolically healthy normal-weight subjects. Similarly, Espinosa et al. [35] showed that weight gain favored the degradation of the metabolically healthy profile, suggesting that obesity outweighs metabolic status in cardiovascular risk. However, our results reveal that at BMI equivalent, metabolically healthy overweight/obese individuals had a lower cardiovascular risk than their metabolically unhealthy counterparts, highlighting the importance of body fat localization. Indeed, studies have shown that metabolically healthy obese people had less visceral and hepatic adipose tissue than metabolically unhealthy obese [36]. Furthermore, Eckel et al. [37] reported that 93.9% of metabolically healthy obese people became unhealthy after 24 years of follow-up, hence the need for regular monitoring to delay this transition, which is influenced in particular by age and certain lifestyle behaviors [38,39].

### Implications for public health policy in Cameroon

From a public health perspective, these results suggest that prevention strategies for cardiometabolic diseases in Cameroon should not rely solely on inflammatory markers such as CRP-hs to identify individuals at risk. Although CRP-hs can detect a state of systemic inflammation at low intensity, its low specificity limits its use as an isolated screening tool. An integrated approach, combining the assessment of adiposity, metabolic parameters, dietary habits, physical activity, and other cardiovascular risk factors, would be more relevant for the early identification of people likely to evolve towards an unhealthy phenotype at the metabolic level. These results argue in favor of strengthening primary prevention interventions targeting health behaviors, including the promotion of a balanced diet, regular physical activity and maintenance of a healthy body weight, in order to prevent progression to cardiometabolic complications.

### Study limitations

This study presents several limitations that must be taken into consideration. First, this study was conducted on an ethnic group of the Cameroonian population and therefore cannot be generalized to the entire population. Secondly, the cross-sectional design of the study limits causal inference. Thirdly, the use of HOMA-IR instead of reference measures such as hyperinsulinemic-euglycemic clamp is a limitation. Fourth, the use of predefined thresholds can lead to some heterogeneity in classification among individuals whose values are close to cut-off points. Fifth, the data from this study did not take into account the distribution of fat in the abdomen, and the evaluation of adiponectin which would have allowed a better explanation of some results.

## Conclusion

In conclusion, the metabolically healthy profile is observed in all categories of BMI within the Cameroonian population. In people with overweight or obesity, it is mainly characterized by an accumulation of gynoid fat, better insulin sensitivity, and reduced cardiovascular risk. These results suggest a metabolic stratification, beyond the BMI, to identify early the people with overweight/obesity most exposed to cardiovascular complications and to move towards personalized prevention and management approaches.
